# Phycocyanin attenuates pulmonary fibrosis via the TLR2-MyD88-NF-κB signaling pathway

**DOI:** 10.1038/s41598-017-06021-5

**Published:** 2017-07-19

**Authors:** Chengcheng Li, Yan Yu, Wenjun Li, Bo Liu, Xudong Jiao, Xinyu Song, Changjun Lv, Song Qin

**Affiliations:** 1 Medicine and Pharmacy Research Center, Binzhou Medical University, Yantai, China; 2 Yantai Institute of Coastal Zone Research, Chinese Academy of Sciences, Yantai, China; 3 Department of Respiratory Medicine, Affiliated Hospital of Binzhou Medical University, Binzhou, China

## Abstract

Our aim was to investigate the effects of phycocyanin (PC) on bleomycin (BLM)-induced pulmonary fibrosis (PF). In this study, C57 BL/6 wild-type (WT) mice and toll-like receptor (TLR) 2 deficient mice were treated with PC for 28 days following BLM exposure. Serum and lung tissues were collected on days 3, 7 and 28. Data shows PC significantly decreased the levels of hydroxyproline (HYP), vimentin, surfactant-associated protein C (SP-C), fibroblast specific protein-1 (S100A4) and α-smooth muscle actin (α-SMA) but dramatically increased E-cadherin and podoplanin (PDPN) expression on day 28. Moreover, PC greatly decreased the levels of interleukin-6 (IL-6), tumor necrosis factor-α (TNF-α) and myeloperoxidase (MPO) at the earlier time. Reduced expression of key genes in the TLR2 pathway was also detected. Compared with WT mice, TLR2-deficient mice exhibited less injury, and the protective effect of PC was partly diminished in this background. These data indicate the anti-fibrotic effects of PC may be mediated by reducing W/D ratio, MPO, IL-6, TNF-α, protecting type I alveolar epithelial cells, inhibiting fibroblast proliferation, attenuating epithelial-mesenchymal transitions (EMT) and reducing oxidative stress. The TLR2-MyD88-NF-κB pathway plays an important role in PC-mediated reduction in pulmonary fibrosis.

## Introduction

Idiopathic pulmonary fibrosis (IPF) is a rare, unexplained, chronic interstitial lung disease with a median survival of 2–3 years. It is generally agreed that IPF commonly arises from a prolonged period of etiological stimulation, abnormal epithelial repair and disordered extracellular matrix deposition. Patients eventually die due to a loss of lung function and respiratory failure^1^. Although many clinical trials for IPF have been attempted, there are few efficient therapies, and the treatments often cause adverse side-effects^2^. Thus, gene targets for effective IPF therapy are urgently needed.

TLRs are receptors involved in pattern recognition and these receptors, especially TLR2, play vital roles in the pathogenesis of various lung diseases^3^. There is extensive evidence that TLRs participate in the process of pulmonary fibrosis by regulating inflammation and wound repair. Liu *et al*.^4^ found that TLR2 mediates the secretion of proinflammatory cytokines and the maturation of dendritic cells (DCs) via the p38-NF-κB and ERK-AP-1 pathways, and the blockade of TLR2 has been reported to markedly reduce BLM-induced pulmonary inflammation and fibrosis in mice^5^. Significant decreases in IL-1, IL-8, TNF-α and macrophage inflammatory protein 2 (MIP-2) were also detected in BLM-treated TLR2^−/−^ mice^6^. In addition to participating in lung inflammation, TLRs are also involved in the induction of EMTs and fibrosis^7^. It was reported that TLR2 contributed to the process of fibrogenesis by regulating the expression of procollagen I/III in heart remodeling^8^. Furthermore, the TLR2-specific agonist Pam3Cys aggravated the formation of fibrosis, but a TLR2 antagonist prevented BLM-induced fibrogenesis^5^. Other TLRs, including TLR3, TLR4, TLR5 and TLR9, are also involved in pulmonary fibrosis^9^. These findings make TLRs attractive targets for the treatment of IPF.

PC, comprising α and β subunits, is a light-harvesting protein found in *Spirulina platensis*. It is a natural edible pigment that participates in algal photosynthesis. Over the last two decades, the health benefits of PC have come to be widely accepted^10^, and PC has been reported to have anti-oxidant^11^, anti-inflammatory^12^ and anti-tumor^13^ activities. Hirahashi *et al*.^14^ found that PC may be the most active component of *Spirulina* extract for improving immunity, and PC can accelerate immune cell proliferation and differentiation by regulating the TLR2/TLR4 signaling pathway. Our previous research revealed that PC extracted from blue green algae could protect rats from paraquat-induced acute lung injury by preventing oxidative damage and inhibiting NF-κB mediated cytotoxicity^15^. PC also protected rats from lipopolysaccharide (LPS)-induced lung injury^16^. However, the underlying molecular mechanisms remain poorly understood. In this study, BLM, the most common treatment used to induce PF in mice, was utilized to examine the role of the TLR2 signaling pathway during the PC-mediated attenuation of PF.

## Results

### PC attenuated BLM-mediated histological changes in mice

**Figure 1:**
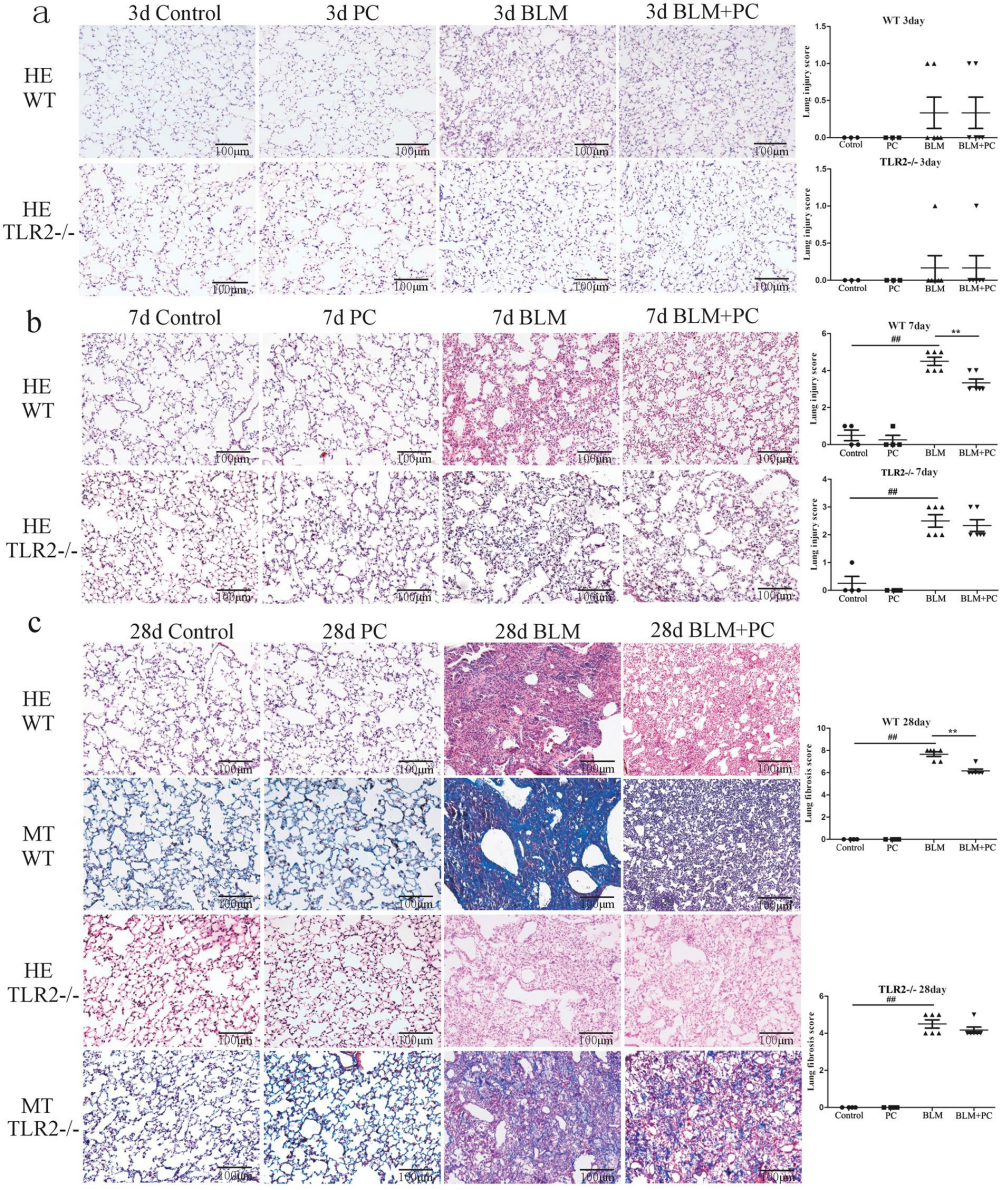
PC attenuated BLM mediated histological changes in mice. Lung tissue sections of control group, PC group, BLM group and BLM + PC group in different phases post-BLM treatment (5 mg/kg) in WT and TLR2^−/−^ mice separately. Severity of lung injury and fibrosis was analysed by the related scoring system. Each bar represents the mean ± SEM of 6–8 mice. ^**##**^p < 0.01 compared with the control group. **p < 0.01 compared with the BLM group. (**a**) HE stain at 3 day. (**b**) HE stain at 7 day. (**c**) HE and Masson stain at 28 day. (HE and Masson’s trichrome stain, magnification 200×).

Lung injury was analyzed by HE and Masson stain. The BLM and BLM + PC groups revealed slight changes in lung tissues 3 days after BLM treatment (Fig. 1a). However, typical histopathological abnormalities, including infiltration of inflammatory cells, an increase in alveolar wall thickness and edema were found in mice 7 days post-BLM treatment (Fig. 1b). Serious damage, e.g., an enlarged alveolar compartment, disordered framework and abnormal collagen deposits, were detected in lung tissues of mice 28 days post-BLM treatment (Fig. 1c). Notably, PC treatment had a significant protective effect against BLM-induced lung injuries at day 7 and day 28. No obvious differences were found between the PC group and the control group. Compared to the injuries induced in the WT mice, fewer injuries were detected in the TLR2^−/−^ mice. And PC-treatment did not have a same strong protective effect in the TLR2^−/−^ mice.

### PC protects against BLM-mediated the high wet/dry (W/D) ratio and MPO activity of lung tissue

**Figure 2:**
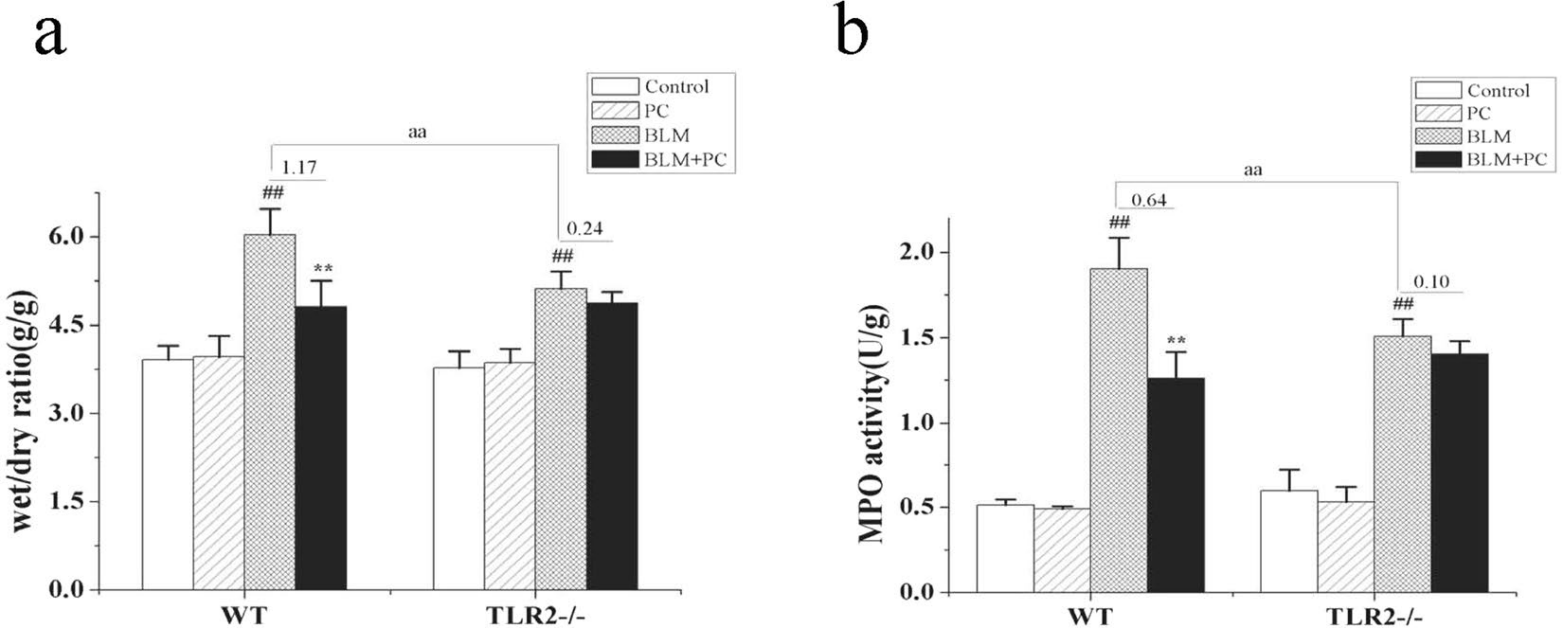
Protective effects of PC on wet/dry ratio (W/D) of lung tissue and MPO activity against BLM treatment. After 7 days intervention, WT and TLR2^−/−^ mice were euthanized, and their lungs were removed. (**a**) Lung W/D ratio. (**b**) MPO activity. Data was represented as the mean ± SEM. (n = 6). ^**##**^p < 0.01 compared with control group, **p < 0.01 compared with the BLM group. ^aa^p < 0.01 compared with WT mice.

The W/D ratio of the lung tissues was evaluated to determine the severity of edema, and the MPO activity was used as a functional index for measuring neutrophil activation^17^. Both measures revealed the degree of lung tissue damage. At 7 days post-treatment, the lung W/D ratios (Fig. 2a) and MPO activities (Fig. 2b) displayed significant increases (p < 0.01) in the WT and TLR2^−/−^ BLM groups. PC treatment significantly reduced these two indices (p < 0.01) in WT mice, but it did not have a protective effect in TLR2^−/−^ mice. These data suggest that PC partially depends on TLR2 to alleviate the lung inflammation in BLM-treated mice.

### The effect of PC on lung HYP, MDA and SOD in BLM mice

**Figure 3:**
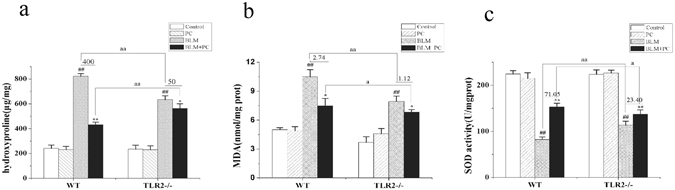
The effect of PC on lung HYP, MDA and SOD in BLM mice. After 28 days intervention, WT and TLR2^−/−^ mice were euthanized, and their lungs were removed. (**a**) HYP content. (**b**) MDA content. (**c**) SOD activity. Data were expressed as mean ± SEM, (n = 6). ^**##**^p < 0.01 compared with the control group, **p < 0.01 compared with the BLM group. ^aa^p < 0.01 compared with WT mice.

The hydroxyproline (HYP) concentration was used to measure collagen deposition/fibrosis in lung tissues of WT and TLR2^−/−^ mice 28 days after BLM treatment. The HYP concentration in TLR2^−/−^ lungs was significantly lower than the concentration in WT lungs (Fig. 3a). The HYP concentrations in tissue from BLM + PC treated WT and TLR2^−/−^ animals were significantly lower than the concentrations in tissue from animals treated with BLM alone. There were no significant differences in HYP concentrations between the control group and the PC group. In addition, BLM treatment increased the malonic aldehyde (MDA) content (Fig. 3b) in tissue from both WT and TLR2^−/−^ mice (p < 0.01), but PC treatment reversed this trend. In tissue from both WT and TLR2^−/−^ mice, the superoxide dismutase (SOD) activity (Fig. 3c) showed opposing tendencies (p < 0.01) in the BLM and BLM + PC groups. The MDA and SOD measurements from the PC group were not significantly different than the measurements from the control group. To summarize, relative to the BLM-induced WT lungs, the BLM-induced TLR2^−/−^ lungs had only mild changes in their MDA and SOD activities, and PC treatment improved these two indices in BLM-induced lungs of either genotype.

### PC increases E-cadherin and decreases vimentin expression during BLM-induced lung fibrosis

**Figure 4:**
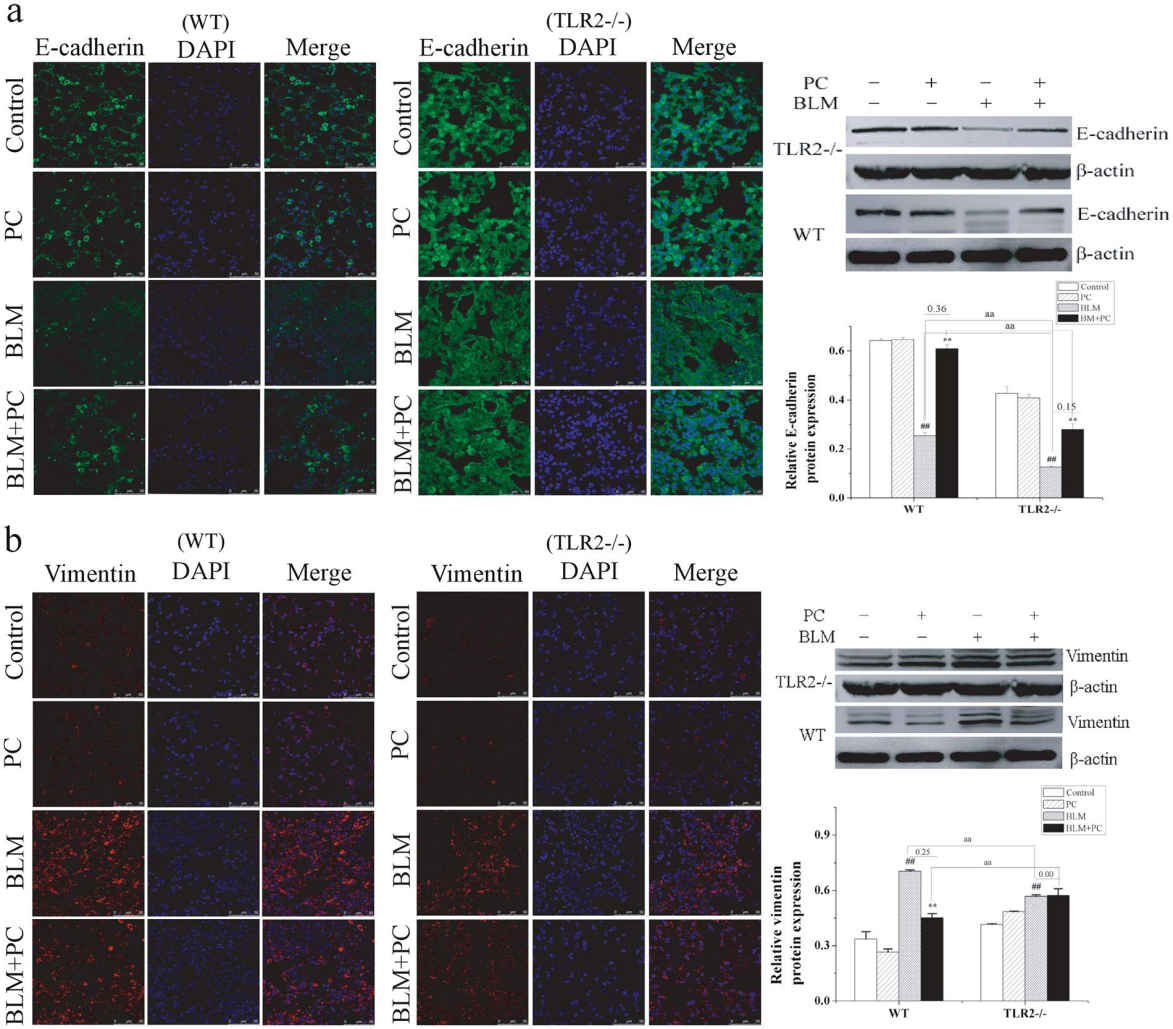
The effect of PC on E-cadherin and vimentin in BLM mice. Following treatment with 5 mg/kg BLM on first day, WT and TLR2^−/−^ mice received treatment for 28 days. Fluorescent immunocytochemistry for DAPI (blue) and (**a**) E-cadherin (green). (**b**) Vimentin (red). Images were visualised under a confocal microscope. Original magnification (400×). Western blot analysis and quantitative densitometry of E-cadherin protein and β-actin reference protein in representative lung tissue on day 28. Images are representative of the three independent studies. Data were expressed as mean ± SEM. ^**##**^p < 0.01 compared with the control group, **p < 0.01 compared with the BLM group. ^aa^p < 0.01 compared with WT mice.

To investigate the expression changes of epithelial and interstitial markers during lung fibrosis, we used the mouse model of BLM-induced lung fibrosis at the 28 day time point. Immunofluorescence and western blot analyses revealed that BLM treatment decreased the levels of E-cadherin and increased the expression of vimentin in lung tissue (Fig. 4a,b), suggesting a decrease or variation in epithelial cells and an increase in interstitial cells during lung fibrosis. PC treatment ameliorated these changes and this activity was partially dependent on TLR2. To identify the cells involved in these changes, we used immunofluorescence to examine the expression levels of PDPN (a marker of type I alveolar epithelial cells)^1^, SP-C (a marker of type II alveolar epithelial cells)^18–20^, S100A4 (a marker of fibroblast)^2^, and α-SMA (a marker of myofibroblasts)^21^ in lung sections at day 28. PDPN expression in uninduced lung epithelial cells was significantly higher compared to expression in the BLM-treated group for both WT and TLR2^−/−^ mice. PC alleviated this damage in the BLM + PC group, especially in WT mice (Fig. S1a). These results were similar to the changes in E-cadherin expression. In contrast to the behavior of PDPN and E-cadherin, many cells expressed SP-C, with absent E-cadherin, in fibrotic lung tissues (Fig. S1b) from BLM-treated WT and TLR2^−/−^ mice. These observations suggested that SP-C may participate in the EMT of lung epithelial cells. PC improved this effect in the WT mice, but no obvious changes were seen in the BLM + PC group in the TLR2^−/−^ mice, implying that TLR2 may participate in the PC-mediated variation of type II alveolar epithelial cells.

In the control and PC groups, S100A4 and vimentin expression were observed within few cells in the interstitium. In WT and TLR2^−/−^ mice treated with BLM, a number of cells in the interstitium expressed S100A4 and high levels of vimentin. Both were decreased only in the WT BLM + PC group (Fig. S2a). The changes in the expression of α-SMA were similar to the changes in the expression of S100A4 (Fig. S2b). These results indicate that PC reduces the activation of fibroblasts in lung tissue and TLR2 participates in this process.

**Figure 5:**
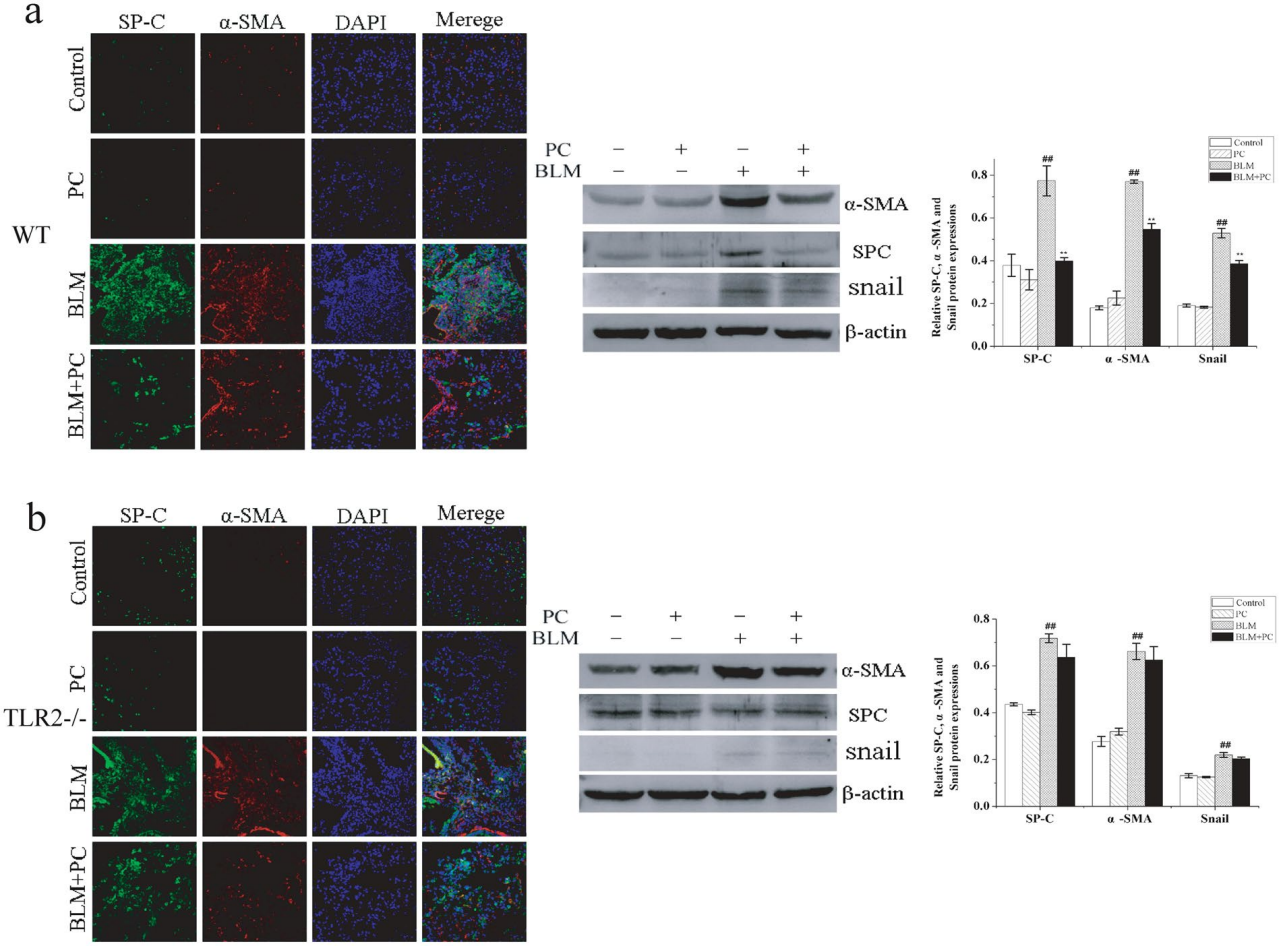
PC alleviated BLM-induced EMT phenomenon. After BLM treatment 28 days, Lung tissue of each group was stained with SP-C and α-SMA. (**a**) WT mice. (**b**) TLR2^−/−^ mice. Fluorescent immunocytochemistry stained for SP-C (green) and α-SMA (red). Images were visualised under a confocal microscope. Original magnification (400×). SP-C, α-SMA and Snail were analyzed by western blot. Data were expressed as mean ± SEM. ^##^p < 0.01 compared with the control group, **p < 0.01 compared with the BLM group. ^aa^p < 0.01 compared with WT mice.

According above results, we speculated that PC could partly inhibit the EMT that occurs during fibrosis. Thus, we performed immunofluorescence using an anti-SP-C antibody and an anti-α-SMA antibody to detect coexpression and quantified the expression of these markers by western blot (Fig. 5a,b). Snail, a well-established master transcriptional regulator of the EMT, was also measured in western blot. Many myofibroblasts and type II alveolar epithelial cells displayed mesenchymal phenotypes in mice fibrotic lung tissues. PC treatment for 28 days significantly alleviated this effect in the WT mice but not in the TLR2^−/−^ mice. These results indicate that PC ameliorates lung fibrosis in part by protecting type I alveolar epithelial cells and inhibiting the EMT. PC also reduces fibroblast activation in lung tissues. These activities may be mediated by TLR2.

### PC blocks TLR2-MyD88-NF-κB signal transduction

**Figure 6:**
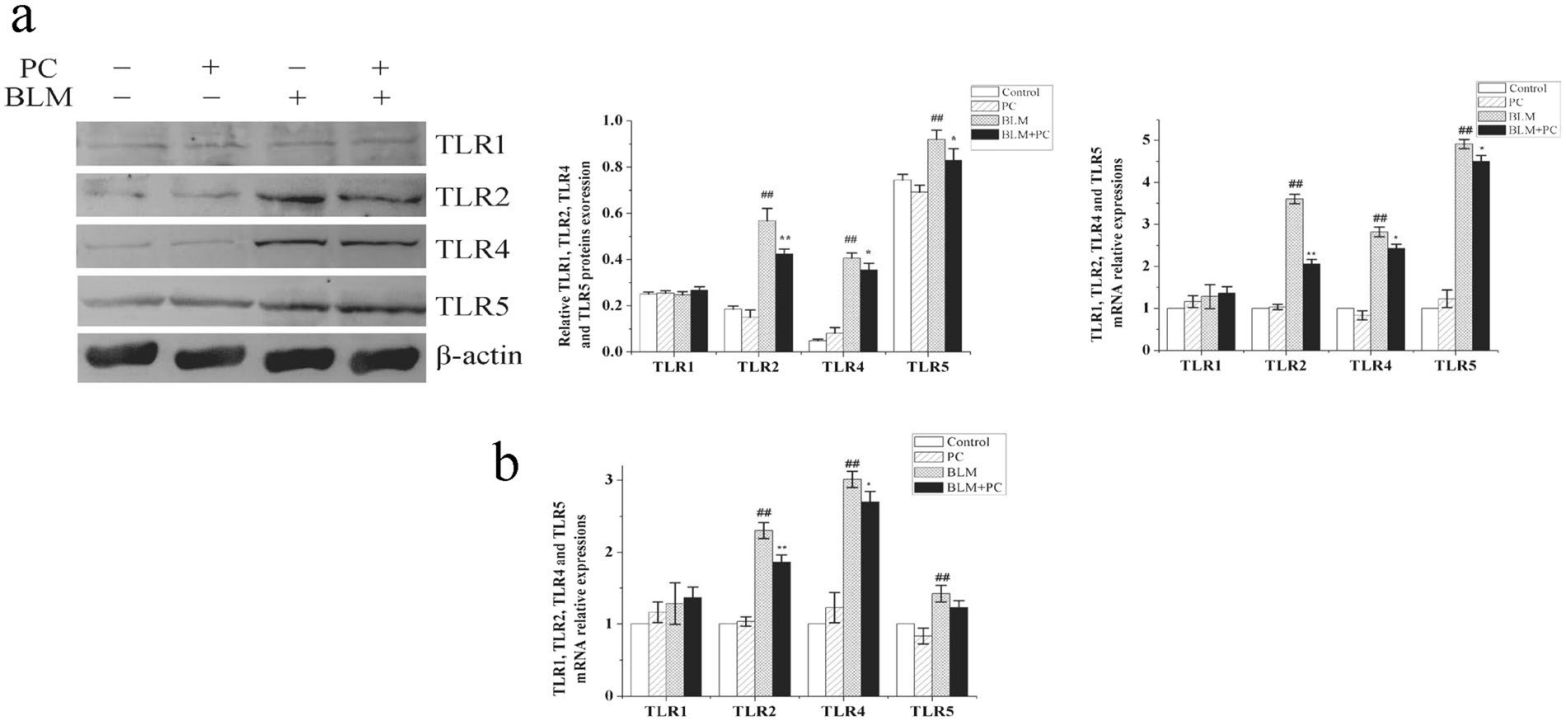
Effects of PC on TLR1, TLR2, TLR4 and TLR5 in the lung tissues induced by BLM. WT mice were killed after BLM exposure for 7 days and 28 days. TLR1, TLR2, TLR4 and TLR5 were analysed in terms of their protein expression levels through Western blot and (or) RT-PCR. (**a**) 7 days. (**b**) 28 days. Data were expressed as mean ± SEM. ^**##**^p < 0.01 compared with the control group, **p < 0.01 compared with the BLM group.

**Figure 7:**
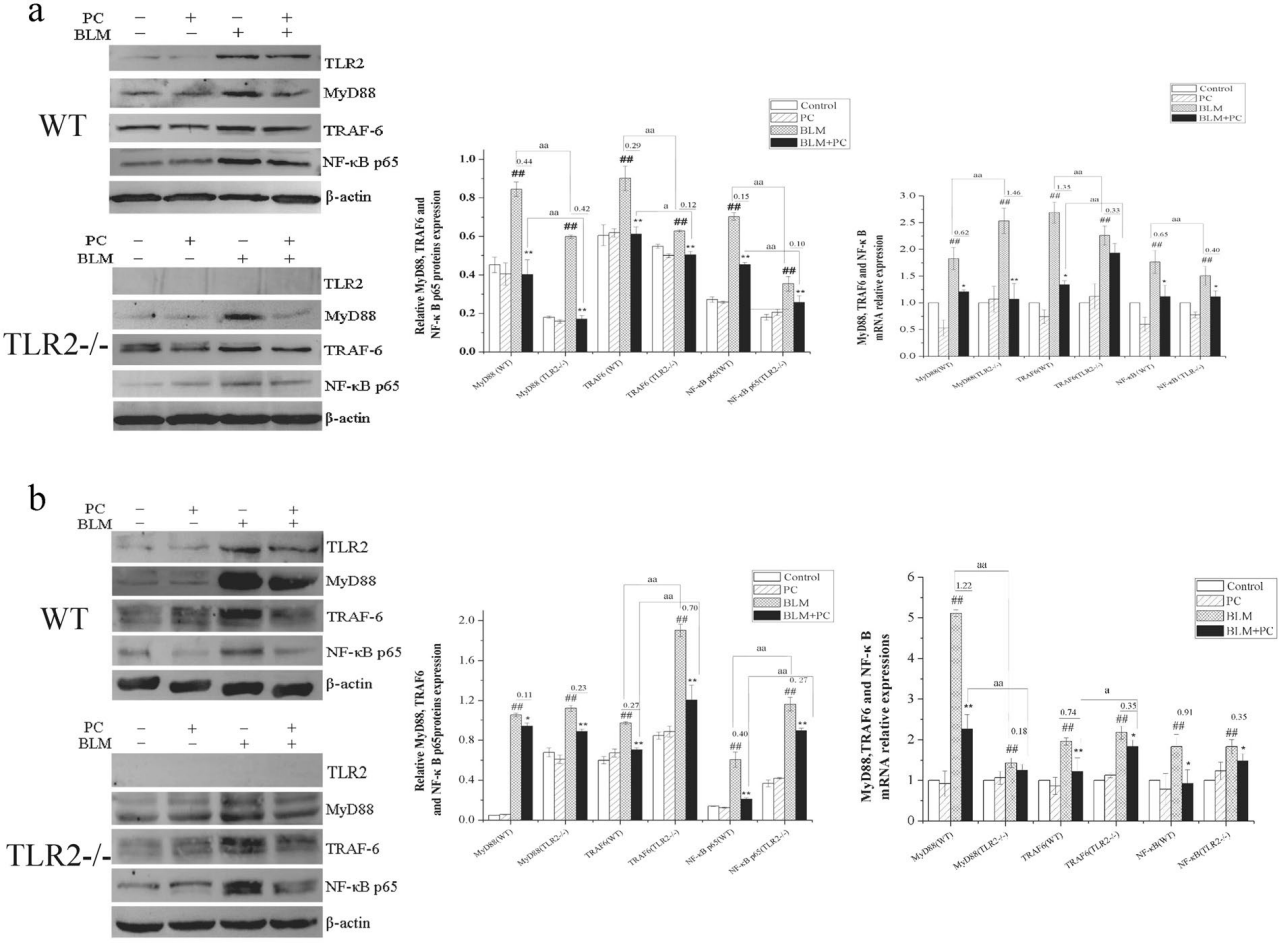
PC blocks TLR2-MyD88-NF-κB signaling transduction. WT and TLR2^−/−^ mice were treated, as described in the text. Mice were killed after BLM exposure at 3 day and 7 day. TLR2, MyD88, TRAF-6 and NF-κB p65 were analysed in terms of their protein expression levels through Western blot and then in terms of β-actin and mRNA relative quantification by using GAPDH. (**a**) 3 day. (**b**) 7 day. Images were representative of the three independent studies. Data were expressed as mean ± SEM. ^**##**^p < 0.01 compared with the control group, **p < 0.01 compared with the BLM group. ^aa^p < 0.01 compared with WT mice.

The activation of TLRs and NF-κB is one of the hallmarks of PF^22^. Previous studies have shown that PC treatment reduced the expression of NF-κB in lung tissues^15^. However, it is unclear whether PC acts via a TLR-dependent mechanism to protect against PF. In a pre-experiment, we assayed the expression levels of several TLRs in the BLM and BLM + PC groups at day 7 (Fig. 6a) and 28 (Fig. 6b). These results revealed that TLR2, TLR4 and TLR5 were significantly increased after BLM stimulation, and PC attenuated these changes, especially the change in TLR2. Therefore, in this paper, we examined the transcriptional and translational levels of the TLR2-MyD88-NF-κB pathway genes by western blot and RT-PCR at day 3 (Fig. 7a), day 7 (Fig. 7b) and day 28 (Fig. S3) in the WT and TLR2^−/−^ mice. Western blot and RT-PCR results confirmed that PC reduced the expression levels of TLR2, MyD88 and NF-κB p65 in BLM-treated mice at day 3 and day 7. Fewer changes in expression were detected on day 28 in all treatment groups using WT mice. These findings indicate that the protective effect of PC against PF partly depends on TLR2, especially at the early stage of the BLM response.

### PC inhibits the BLM-induced release of cytokines

**Figure 8:**
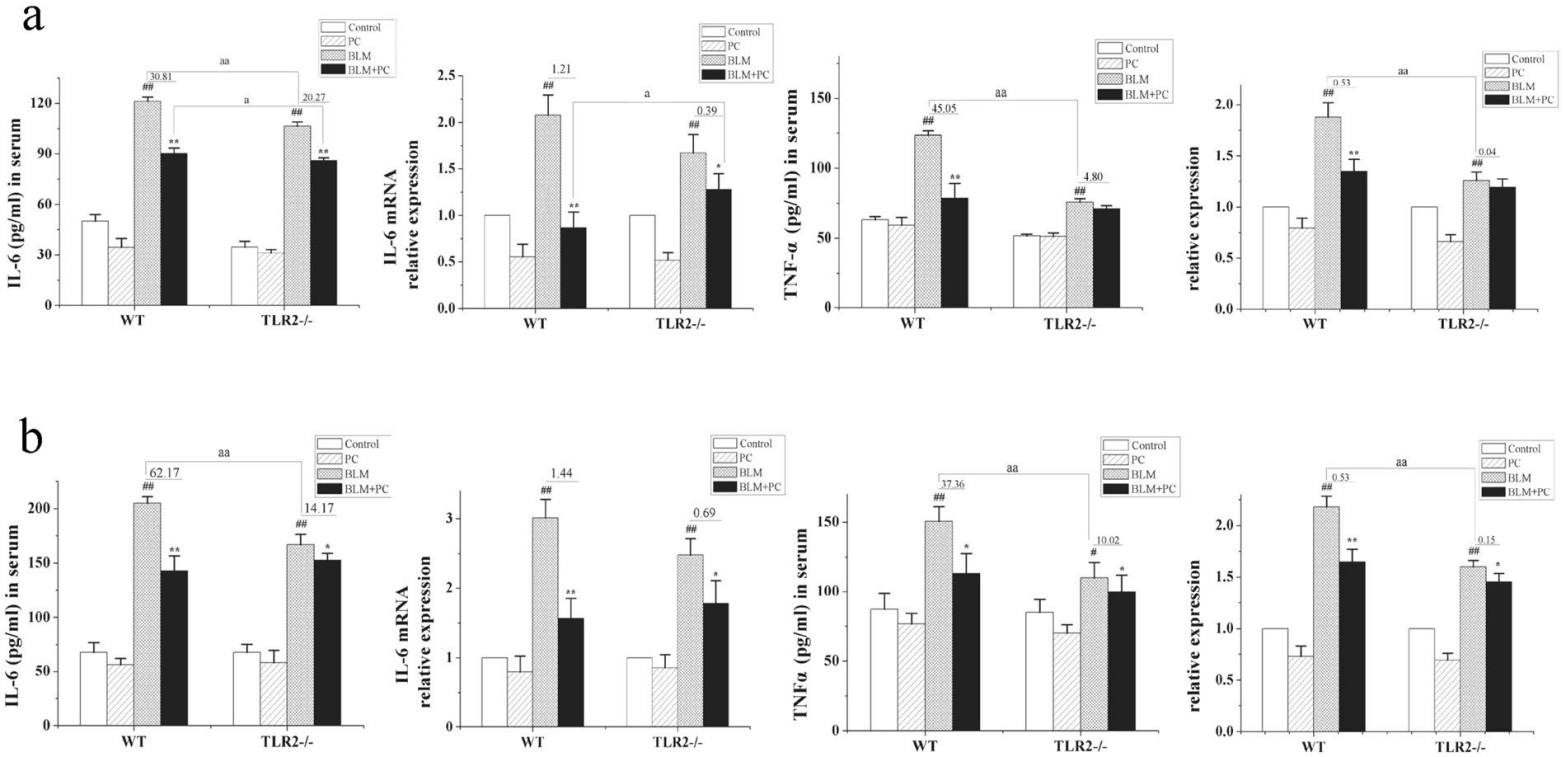
PC inhibits BLM-induced cytokines release. WT and TLR2^−/−^ mice were treated as described in the text. Mice were killed after BLM exposure at 3 day and 7 day. IL-6 and TNF-α levels in serum were measured through ELISA. mRNA levels were also observed in lung fibrosis. (**a**) 3 day. (**b**) 7 day. Data were expressed as mean ± SEM, (n = 6). ^**##**^p < 0.01 compared with the control group, **p < 0.01 compared with the BLM group. ^aa^p < 0.01 compared with WT mice.

Migration of inflammatory cells into alveolar spaces is another crucial characteristic in lung injury^23^, among which neutrophils are the major cells. Upon activation, the secretion of IL-6 and TNF-α were ascending, which amplify the inflammatory responses and participate in the pathological injuries^24^. On day 3, the levels of IL-6 and TNF-α in the serum of the WT and TLR2^−/−^ mice were compared by ELISA (Fig. 8a). We also examined the expression levels of these two cytokines in lung tissues using RT-PCR at the same stage (Fig. 8a). ELISA and RT-PCR results revealed that the expression levels of IL-6 (p < 0.01) and TNF-α (p < 0.01) in serum and lung tissues were higher in the BLM group compared to the control group on day 3. The IL-6 expression levels in the serum and lung tissues of the WT and TLR2^−/−^ mice were decreased in the BLM + PC group (p < 0.01). However, PC treatment only decreased the expression levels of TNF-α in the serum and lung tissue from BLM-treated WT mice but not from BLM-treated TLR2^−/−^ mice. No obvious changes in IL-6 or TNF-α expression were observed between the control group and the PC group. The IL-6 and TNF-α levels in serum and lung tissue were also measured on day 7 (Fig. 8b). On day 7, the levels of IL-6 (p < 0.01) and TNF-α (p < 0.01) were significantly increased in BLM-treated WT mice, and PC treatment decreased the expression levels of these inflammatory cytokines. TLR2 deficiency influenced the therapeutic efficacy of PC, which suggests that TLR2 plays a partial role in the anti-inflammatory effects of PC.

## Discussion

This study demonstrated that PC alleviates BLM induced pulmonary injury and fibrosis by inhibiting the TLR2-MyD88-NF-κB pathway at the early stage and attenuates the EMT during the fibrotic process.

BLM is extensively used for mechanistic investigation of IPF in mice and causes a time-dependent increase in tissue-infiltrating proinflammatory cells and cytokines^25^. In our study, IL-6 and TNF-α levels in serum increased 3 days after BLM instillation. By day 7 post-BLM exposure, the W/D ratio, MPO concentration and levels of inflammatory factors were markedly increased, and many inflammatory cells had infiltrated the lung tissue. Notably, PC significantly inhibited these immune responses in the BLM treated mice. Similarly, a former study revealed that PC reversed the increase in MPO concentration, lung W/D ratio and NF-κB expression following LPS-induced acute lung injury^16^. Shih *et al*. reported that PC significantly inhibited TNF-α, IL-1β, IL-6 secretion and neutrophil infiltration in inflammatory sites resulting from experimental hyperalgesia^26^. The increased levels of TNF-α, IL-1β and IL-6 after LPS stimulation in microglial cells could also be reversed by PC^27^. These results suggest that PC has a strong property to inhibit inflammatory cytokines release that alleviates multiple conditions, including BLM-induced lung injury.

Recently, it was reported that membrane-expressed TLRs are involved in many acute or chronic lung diseases. Chen *et al*. found that TLR4 participates in the process of acute lung injury^28^, TLR2 and TLR4 are related to the formation of chronic obstructive pulmonary disease^29^, and TLR1 is capable of binding mycobacterial molecules during the occurrence of pulmonary tuberculosis^30^. TLR2, TLR4 and TLR5 are most likely involved in the recognition of bacteria in the lungs, and TLR5 is also present on the airway epithelial surface^31^. These studies confirmed that TLRs, especially TLR2 and TLR4, are implicated in PF^32^. In our preliminary study, TLR2 was involved in the protective effect of PC against BLM-induced PF. However, the detailed mechanism remains unclear. Several studies reported that TLR2 played an important role in the development of IPF. TLR2 deficiency reduced pulmonary inflammatory response and subsequently attenuated fibrosis^5^ through regulating inflammatory immune responses. The present results revealed that PC reduced the expression levels of key genes related to the TLR2-MyD88-NF-κB pathway that were normally elevated on day 3 and day 7 post-BLM treatment. Interestingly, TLR2 deficiency partly attenuated the protective effects of PC. In summary, the TLR2 pathway plays a crucial role during the early inflammatory stage of PF, and the inhibition of the TLR2 pathway may be integral to the protective effect of PC.

PF involves the replacement of normal lung structure with fibrotic tissue accompanied by epithelial damage, fibroblast proliferation, and excessive extracellular matrix deposition. We found that PC clearly attenuated the BLM-induced upregulation of vimentin and downregulation of E-cadherin at day 28 post-BLM treatment in WT mice. It is reported that the EMT of epithelial cells to myofibroblasts plays a vital role in the pathogenesis of pulmonary fibrosis^33^. Alveolar epithelial cells gradually lose epithelial cell markers, express mesenchymal markers and acquire single-cell motility during the development of PF^34^. These phenomena were observed in this study. PC not only protected type I alveolar epithelial cells and reduced lung fibroblast proliferation but also partly inhibited the EMT of lung tissues. These activities may involve TLR2. However, the expression of TLR2 was shown to increase until 7 days post-BLM exposure and then decrease with further BLM exposure^4^. We obtained similar results. This suggests that the PC-induced inhibition of the TLR2 pathway plays an important role during the early stages of lung fibrogenesis. Additional immune related genes, including Toll-like/IL-1β receptors, IL-6 and IL-10 are implicated in various EMT-induced diseases^35^. In our study, PC significantly inhibited the TLR2-MyD88-NF-κB pathway and reduced IL-6 secretion 3 days and 7 days post-BLM treatment. That maybe an inhibit mechanism of EMT. A number of studies also revealed that intracellular cascades, mTOR/PI3K/Akt34^36^, MAPK/JNK^37^, Rho GTPases^38^ are also the core mediators transducing signals to activate alveolar epithelial cells type II. PC could promote autophagic cell death by inhibiting PI3/Akt/mTOR signaling pathways in pancreatic adenocarcinoma^39^. And PC also inhibits PI3K and Akt in 1,2-Dimethylhydrazine-induced colon cancer in mice^40^. Subsequently, PC significantly attenuated the BLM-induced EMT in pulmonary tissue at day 28. These results suggest that PC may alleviate the BLM-induced EMT by inhibiting the TLR2 signaling pathway and IL-6 secretion at the early stages of PF.

Oxidative stress has been associated with acute and chronic lung disorders, including IPF^41^. Our previous paper also verified that PC protects against PF via an anti-oxidant effect, but the underlying mechanism is unclear. The reactive oxygen species (ROS) generated by BLM intratracheal instillation directly injure lung cells and contribute to extracellular matrix deposition^42^. In our study, PC significantly reduced the changes in ROS-related markers, including MDA content and SOD activity in WT and TLR2^−/−^ mice. This finding indicates that PC has potent anti-oxidant activity, which is only a small part dependent on the TLR2 signaling pathway. Many studies have shown that PC has a strong anti-oxidant effect *in vitro* and *in vivo* ^43^. The anti-oxidative effect of PC may be associated with its tetrapyrrol structure^44^.

In summary, the present study demonstrates that the anti-fibrotic effects of PC may be mediated by alleviating IL-6 and TNF-α release, protecting type I alveolar epithelial cells, inhibiting fibroblast proliferation, attenuating epithelial-mesenchymal transitions (EMT). The TLR2-MyD88-NF-κB signaling pathway plays an important role in above processes.

## Materials and Methods

### Reagents

PC (Xindaze Spirulina Co. Ltd, China). Anti TLR2 antibody, anti MyD88 antibody, anti NF-κB p65 antibody, anti S100A4 antibody and anti α-SMA antibody (Abcam, UK). Anti-SP-C antibody and anti-PDPN antibody (Santa Cruz). Anti-TRAF-6 antibody and anti-TLR4 antibody (Proteintech, China). Anti-TLR1 antibody and anti-TLR5 antibody (NOVUS, USA). BLM (Nippon Kayaku, Japan). Kits IL-6 and TNF-α enzyme-linked immunosorbent assay kits (Sigma, USA). MPO (No. A044), Malondialdehyde (MDA) (A003-1), HYP (A030-2), Masson and Superoxide dismutase (SOD) (A001-3) kits (Nanjing Jiancheng Bioengineering Institute, China).

### Animals

6 to 8-week-old, specific pathogen free grade, SCXK2015-0001, male C57 BL/6 WT mice (n = 120) and male TLR2^−/−^ mice (n = 120) (Model Animal Research Center of Nanjing University, China) were maintained at the Binzhou Medical University. Mice in all groups were housed in a temperature controlled environment (26 °C) with 12 h/12 h light/dark cycles and *ad libitum* energy intake. All animals were allowed one week to acclimate prior to experimentation. They were handled according to the guidelines and regulations of the Canadian Council on Animal Care. The protocol was approved by the Committee on the Ethics of Animal Experiments of Binzhou Medical University (Permit no. SCXK20140005).

### Induction of PF by BLM

Several lines of research have proven that a single tracheal instillation of BLM causes PF in mice^45^. In our study, mice were randomly divided into four groups: the control group, PC group, BLM group and BLM + PC group. After an overnight fast^46–49^, PF mice were anesthetized and induced with 5 mg/kg BLM dissolved in 50 μl of sterile saline via a single intratracheal instillation. The mice in the control and PC groups received equal volumes of saline. After 6 h, mice in the PC group and the BLM + PC group were administered 50 mg/kg PC daily (the dose was selected from the pre-experiment). On day 3, day 7 and day 28, mice were anaesthetized and sacrificed. Serum and lung tissues were collected from mice and stored in liquid nitrogen until use.

### Histologic examination

The histological examination was performed using light microscopy. In our study, a single left lung lobe per mouse was perfused, fixed in 4% formaldehyde, and embedded in paraffin according to the standard procedure. Serial 5-μm sections were stained with hematoxylin and eosin (HE) and Masson’s trichrome (MT). The sections were imaged using an IMAGE-PRO PLUS microscope (Media Cybernetics, USA). Lung injury scores were assessed according to previous methods^50^. In brief, a score of 0, 1, 2, 3 and 4 represents no damage, mild damage, moderate damage, severe damage and very severe histologic changes, respectively. In addition, 28 days after BLM treatment, fibrosis was quantified using the modified Ashcroft scoring^51^.

### Wet-to-dry (W/D) weight ratio and MPO activity in lung tissues

Pulmonary edema is the hallmark of acute lung injury, enhanced vascular permeability is one of typical symptoms of acute inflammatory responses not only in systemic inflammation but also in in lung tissue. W/D ratio was evaluated as an index of pulmonary edema. Seven days after the BLM challenge, several mice in each group were euthanized, and their right lungs were excised and rinsed with saline to remove blood. The lower lobe of the right lung was blotted dry, weighed, and placed in an oven at 60 °C for 72 h to obtain the ‘dry’ weight. MPO activity was measured by precisely following the operational manual. Briefly, lung tissue (approximately 30 mg) was homogenized and fluidized to obtain a 5% homogenate in extraction buffer. Then, 0.45 ml homogenate was mixed with 0.05 ml reaction buffer and heated to 37 °C in a water bath for 15 min. The enzymatic activity was determined by measuring the changes in absorbance at 460 nm using an Ultra Multiskan Ascent.

### Measurement of HYP, MDA and SOD activity in lung tissues

Twenty-eight days after BLM treatment, lung tissues were removed and weighed, a four-fold volume of normal saline was added and the tissues were homogenized using an ultrasonic JYD-900 (Shanghai Zhixin Instrument Co., Ltd, China) operated at 200 Watts with the following cycle: work 8 sec, free 15 sec, repeat 8 times. After 30 min incubation on ice, samples were centrifuged at 1200 r/min at 4 °C for 20 min, and the supernatants were used for detecting MDA and SOD. MDA content was determined by the thiobarbituric acid method^52^, whereas SOD activity was evaluated according to the xanthine oxidase method as described previously by Yao^53^. Lung HYP content was measured based on alkaline hydrolysis of the lung tissues to assess collagen deposition. The necessary total protein concentrations were measured by the BCA protein assay kit (Solarbio, Beijing, China).

### Determination of serum IL-6 and TNF-α levels in PF mice

Concentrations of cytokines were determined by ELISA. For measuring serum IL-6 and TNF-α, the ELISA SIGMA-ALDRICH^®^ Kit was used following the manufacturer’s instructions, and the optical density of each well was obtained at 450 nm by the Ultra Multiskan Ascent (Thermo Labsystems, USA).

### Western blot analysis

Lung tissues were suspended in RIPA lysis buffer with 1 mmol/L protease inhibitor cocktail (Solarbio, Beijing, China), homogenized, and clarified by centrifugation at 12000 r/min for 10 min at 4 °C. Protein amounts were quantified by the BCA method, and supernatants were mixed with loading buffer; equal amounts of the proteins (approximately 50 µg) were separated by SDS-PAGE (8% to 12% gels) and subsequently transferred to 0.22 μm polyvinylidene difluoride membranes (Millipore). After transfer, membranes were first blocked for 3 h in TBST (0.1% Tween-20 in 1× TBS, pH 7.4) buffer containing 5% (w/v) nonfat milk powder at room temperature, followed by incubation overnight at 4 °C with the appropriate specific primary antibodies against TLR1 (1/1000), TLR2 (1/1000), TLR4 (1/1000), TLR5 (1/1000), MyD88 (1/1000), NF-κB p65 (1/8000), TRAF6 (1/500) and β-actin (1/1500; Bioss, Wuhan, China) diluted in TBST buffer. After washing, membranes were incubated in the appropriate secondary antibodies for 1 h at room temperature. The immunoblots were developed using ECL (Novland, Shanghai), scanned with a ChemiScope Mini 3100 (Clinx, China) and quantified using Image J software.

### Immunofluorescence

E-cadherin, vimentin, PDPN, SP-C, S100A4 and α-SMA were analyzed by immunofluorescence. Briefly, paraffin sections were incubated for 30 min at 60 °C and treated with xylene and a graded ethanol series to remove paraffin and rehydrate. After rinsing 3 times with PBS, the slices were permeabilized with 0.1% Triton X-100 in PBS at room temperature for 8 min. Then, for antigen retrieval, the lung sections were heated in EDTA antigen repair solution (Beyotime, P0085) in a microwave (800 W, 8- to 10-minute cycles). After 2 h, the sections were incubated in goat serum blocking solution for 30 min at room temperature then immunostained with primary antibodies against E-cadherin (CST, 1/50), vimentin (Abcam, 1/1000), PDPN (1/50). SP-C (1/50), S100A4 (1/250) or α-SMA (1/100) overnight at 4 °C followed by incubation with Fluorescein-conjugated AffiniPure goat anti-mouse IgG (H + L) (1/200), DyLight-549 labeled anti-rabbit IgG (1/200), DyLight-488 conjugated rabbit anti-goat anti-rabbit IgG (H + L) (1/200), or DyLight-649 Conjugated rabbit anti-goat anti-rabbit IgG (H + L) (1/200) for 1 h at room temperature. After secondary antibody incubation, nuclei were stained with DAPI (66.7 μg/ml) for 5 min. After washing with PBS, coverslips were covered with anti-fade solution (Solarbio) and placed onto the slides. The slides were analyzed by confocal microscopy (LEICA TCS SPE).

### Quantitative RT‑PCR

Total RNA from the lung tissues was extracted using Trizol reagent (Takara Bio, Dalian, China). The purity and quality of the RNA samples were examined with an ultraviolet spectrophotometer at 260 and 280 nm. Then, 2 μg of total RNA was reverse transcribed into cDNA; 2 μl cDNA from each sample was amplified in a 25 µl PCR reaction (Takara Bio, Dalian, China) containing 12.5 µl 2× SYBR Premix Ex Taq (Tli RNaseH Plus) and 0.4 µM of each primer. Quantitative RT-PCR was performed on a BIO-RAD RT-qPCR system (iQ5 Optical Module, USA). After an initial denaturation at 95 °C for 30 sec, the samples were subjected to 40 amplification cycles of denaturation at 95 °C for 5 sec and annealing at 60 °C for 30 sec. After amplification, a melt curve analysis was performed. GAPDH was used as an internal control for sample loading and integrity. The details of the primers (Invitrogen, China) are listed in Table 1. All reactions were performed in triplicate, and targeted transcript levels were calculated and normalized to the transcript level of a housekeeping gene (GAPDH). Quantitative Real-time PCR results were calculated using the 2^−∆∆CT^ method as previously described^54^.

**Table 1:** Primers used for quantitative real-time RT-PCR.

Primers		Sequence (5′ → 3′)
TLR2	Forward	CTC AGC GAA AAT CTG ATG GT
	Reverse	TCA AAT GAT TCT GGC TCA AAA
MyD88	Forward	TGG TGG TTG TTT CTG ACG AT
	Reverse	GGA AAG TCC TTC TTC ATC GC
TRAF-6	Forward	ATG AGT CTC TTA AAC TGT GAG AAC AGC
	Reverse	CTA CAC CCC CGC ATC AGT ACT T
NF-κB	Forward	GCT ACA CAG AGG CCA TTG AA
	Reverse	TCC CGG AGT TCA TCT ATG TG
IL-6	Forward	AAA TGA TGG ATG CTA CCA AAC T
	Reverse	CCA GAA GAC CAG AGG AAA TTT T
TNF-α	Forward	AGG CAC TCC CCC AAA AGA T
	Reverse	CAG TAG ACA GAA GAG CGT GGT G
GAPDH	Forward	CAG TGG CAA AGT GGA GAT TG
	Reverse	CGT TGA ATT TGC CGT GAG T

### Statistical analysis

Data were analyzed for statistical significance using SPSS 13.0 software. The data were expressed as the means ± standard deviations (SDs). Comparisons between groups were performed using the test of homogeneity of variances and one-way analysis of variance (ANOVA). Unpaired Student’s t-test was used for experiments comparing the WT and TLR2^−/−^ groups; p < 0.05 and p < 0.01 were considered statistically significant.

## Electronic supplementary material

supplementary info
