# Supplementary material for: Phycocyanin attenuates pulmonary fibrosis via the TLR2-MyD88-NF-κB signaling pathway
Source: Sci Rep. 2017 Jul 19;7:5843. doi: 10.1038/s41598-017-06021-5 (PMC5517415; doi:10.1038/s41598-017-06021-5)
Supplement: Supplementary file 1 — supplementary info [file 41598_2017_6021_MOESM1_ESM.pdf]

Phycocyanin attenuates pulmonary fibrosis via the TLR2-MyD88-NF-κB signaling pathway

Chengcheng Li[1], Yan Yu[1], Wenjun Li[2], Bo Liu[1], Xudong Jiao[2], Xinyu Song[1], Changjun Lv[3], Song Qin[2]

Chengcheng Li and Yan Yu contributed equally to this study.

Affiliation

1 Medicine and Pharmacy Research Center, Binzhou Medical University, Yantai, China

2 Yantai Institute of Coastal Zone Research, Chinese Academy of Sciences, Yantai, China

3 Department of Respiratory Medicine, Affiliated Hospital of Binzhou Medical University, Binzhou, China

Corresponding author

Prof. Changjun Lv, Department of Respiratory Medicine, Affiliated Hospital of Binzhou Medical University, Huanghe 2nd Road, Binzhou, Shandong, China, 256603 Tel: +86-543-3258728, E-mail: lucky_lcj@sina.com

Prof. Song Qin, Yantai Institute of Coastal Zone Research, Chinese Academy of Sciences, 17 Chunhui Road, Yantai, Shandong, China, 264003, E-mail: cpra_yic@sina.com

Lv CJ and Qin S conceived the idea and participated in the design of the study. Li CC, YU Y and Li WJ designed and performed the experiments, and Li CC and YU Y contributed equally to this study. Li CC prepared the manuscript, and Liu B, Jiao XD, Song XY, Lv CJ and Qin S read and approved the final manuscript.

Competing financial interests

The authors declare no competing financial interests.

Supplementary information

Figure S1

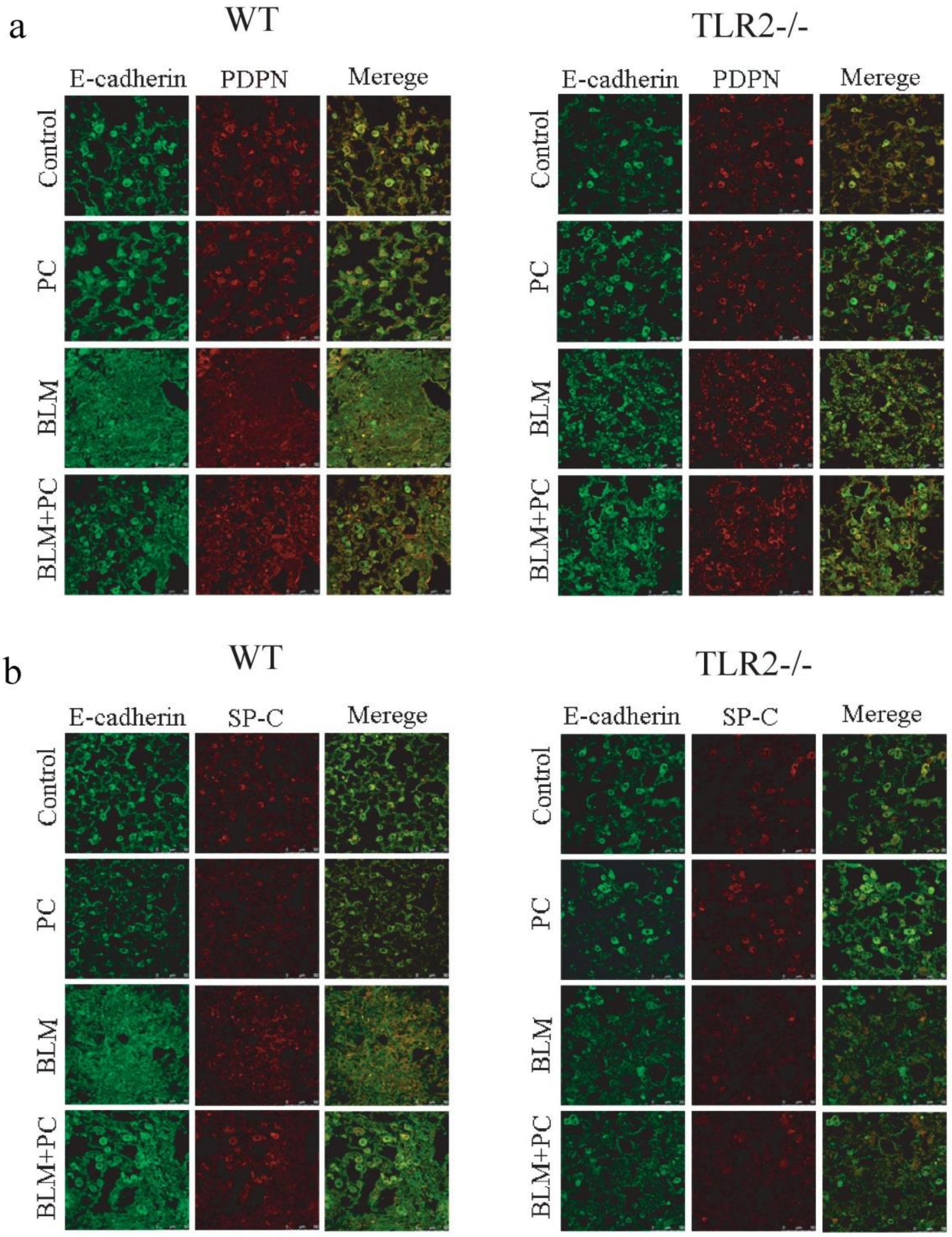

Figure S1. PC protected type I alveolar epithelial cells and alleviated type II alveolar epithelial cells differentiation. After BLM treatment 28 days, Lung tissue of each group was stained with (a) E-cadherin (green) and PDPN (red). (b) E-cadherin (green) and SP-C (red). Fluorescent immunocytochemistry stained for Images were visualised under a confocal microscope. Original magnification (400×).

Figure S2

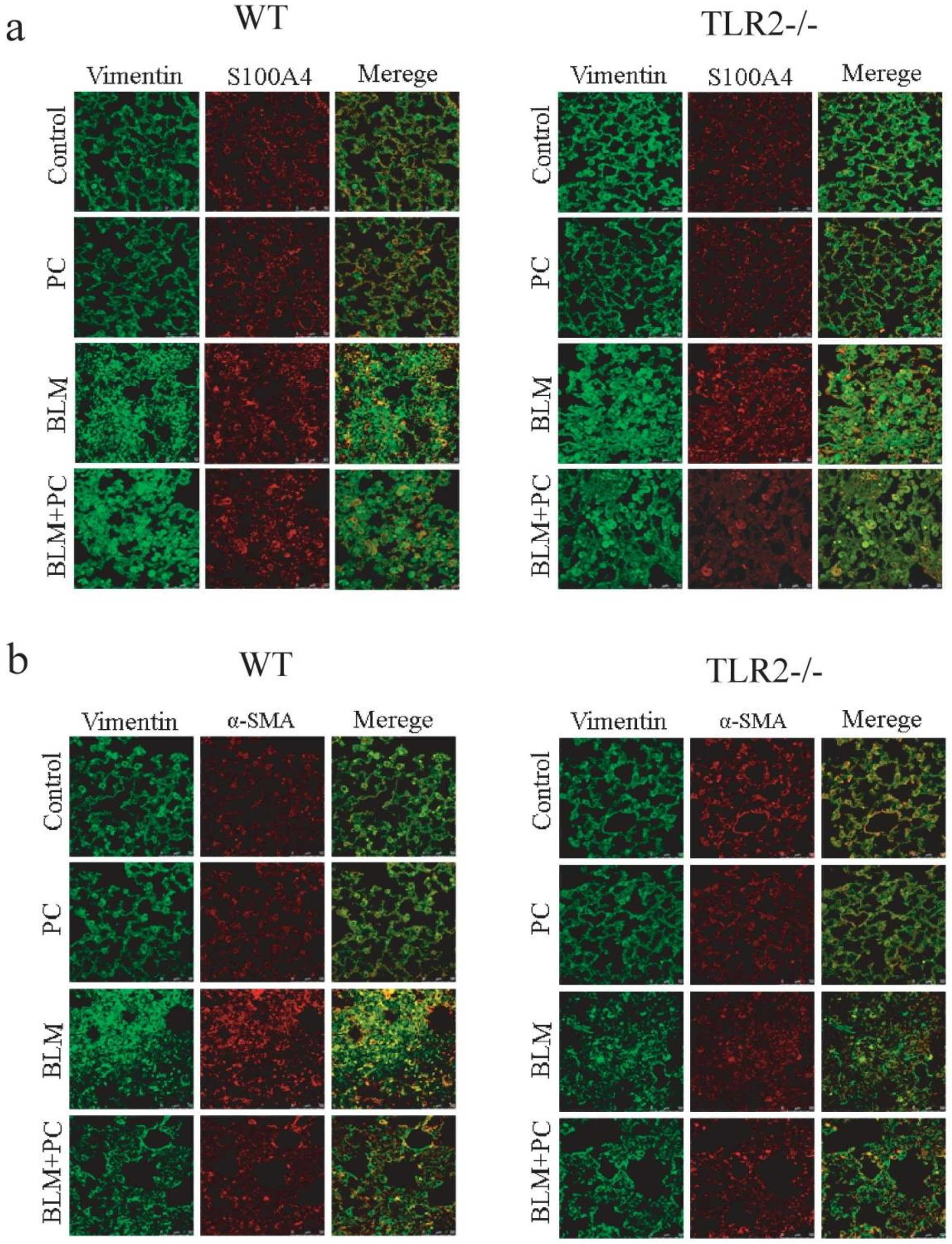

Figure S2. PC alleviated aberrant proliferation of fibroblasts and the activation of myofibroblasts. After BLM treatment 28 days, Lung tissue of each group was stained with (a) Vimentin (green) and S100A4 (red). (b) Vimentin (green) and α-SMA (red). Fluorescent immunocytochemistry stained for Images were visualised under a confocal microscope. Original magnification (400×).

Figure S3

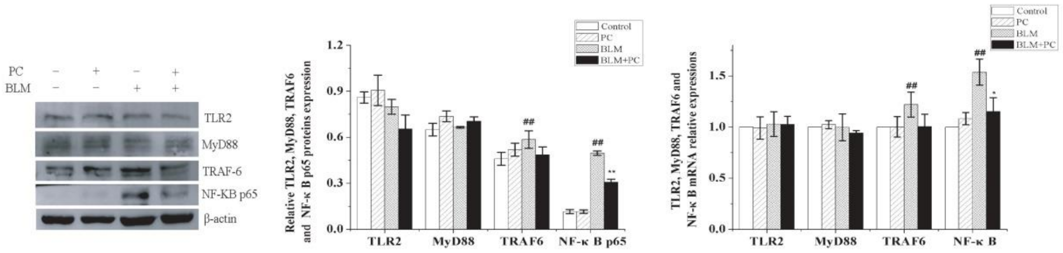

Figure S3. PC's influence over TLR2-MyD88-NF-κB signaling transduction. WT mice were treated as described in the text. Mice were killed after BLM exposure 28day. TLR2, MyD88, TRAF-6 and NF-κB p65 were analysed in terms of their protein expression levels through Western blot and then in terms of β-actin and mRNA relative quantification by using GAPDH. Images were representative of the three independent studies. Data were expressed as mean ± SEM. $^{\#\#}p < 0.01$ compared with the control group, $^{**}p < 0.01$ compared with the BLM group.
